# Disentangling diatom species complexes: does morphometry suffice?

**DOI:** 10.7717/peerj.4159

**Published:** 2017-12-11

**Authors:** Saúl Blanco, María Borrego-Ramos, Adriana Olenici

**Affiliations:** 1 Institute of the Environment, Leon, Spain; 2 Faculty of Environmental Sciences and Engineering, Babes-Bolyai University of Cluj-Napoca, Romania

**Keywords:** Taxonomy, Biometry, Automated identification, Classification, Multivariate statistics

## Abstract

Accurate taxonomic resolution in light microscopy analyses of microalgae is essential to achieve high quality, comparable results in both floristic analyses and biomonitoring studies. A number of closely related diatom taxa have been detected to date co-occurring within benthic diatom assemblages, sharing many morphological, morphometrical and ecological characteristics. In this contribution, we analysed the hypothesis that, where a large sample size (number of individuals) is available, common morphometrical parameters (valve length, width and stria density) are sufficient to achieve a correct identification to the species level. We focused on some common diatom taxa belonging to the genus *Gomphonema*. More than 400 valves and frustules were photographed in valve view and measured using Fiji software. Several statistical tools (mixture and discriminant analysis, k-means clustering, classification trees, etc.) were explored to test whether mere morphometry, independently of other valve features, leads to correct identifications, when compared to identifications made by experts. In view of the results obtained, morphometry-based determination in diatom taxonomy is discouraged.

## Introduction

Diatoms are unicellular algae inhabiting many different aquatic and terrestrial environments worldwide. To date, ∼10^5^ different species have been described (Mann & Droop, 1996), with particular ecological preferences, so that there is a clear relationship between diatom communities and the environmental characteristics of their habitats. The reliability of diatom-based biomonitoring methods has long been established, but diatom analyses are also useful in palaeoecology, biotechnology or forensic applications (Stoermer & Smol, 2001). However, the main obstacle limiting their use lies in the difficulty of their taxonomic identification, since diagnoses at specific or subspecific levels are often required. This implies important investments in optical equipment and expert training. Currently, the identification and routine counting of diatoms is performed under light/phase contrast optical microscopy, but several tools are being proposed to automate the identification process by means of image analyses (Buf & Bayer, 2002; Kloster, Kauer & Beszteri, 2014) or DNA metabacoding (e.g., Vasselon et al., 2017).

Diatom cell size (length [L], width [W], L/W ratio) and other morphometric parameters (e.g stria density [S]) are commonly used in taxonomic keys aiding identification, together with shape features and ultrastructural characteristics. Despite the fact that basic morphometric insights are of fundamental importance to diatom taxonomy (Beszteri, Ács & Medlin, 2005), the use of quantitative approaches in diatom identification is relatively rare (Beszteri, 2005) and taxa identification is mostly based on cell shape, which is considered to be stable on a large scale (Mou & Stoermer, 1992). Due to their reproductive cycle, there is a tendency within each species to decrease in cell size after several generations so that natural populations exhibit broad and often skewed size distributions, since they are made up of several age classes (Spaulding et al., 2012). This cyclical change in size can vary also between populations (Pappas & Stoermer, 2003) and is often accompanied by an allometric change in the L/W ratio (Schmid, 1994). Some studies suggest also a dependence of morphometric parameters on habitat features (Cortese & Gersonde, 2007).

Diatom taxa are often arranged in groups of morphologically similar forms that share numerous features and with overlapping morphometric ranges (Potapova & Hamilton, 2007). A number of “species complexes” have been reported with a high level of diversity (Ajani et al., 2013). These complexes may occur in sympatry (Kulichová & Fialová, 2016), so that several taxa can be found in a single diatom community. The resolution of morphologically similar taxa is especially difficult for microscopy analyses, and misidentifications can lead to inaccurate environmental inferences. In this paper, we explore the possibility of disentangling one of these groups (species related to *Gomphonema gracile* Ehr. (Reichardt, 2015) and *G*. *parvulum* (Kütz.) Kütz.) by unsupervised classification of individuals based only on their morphometric parameters. We tested a number of classification algorithms and compared their result with identifications made by experts under light microscopy (LM). Our hypothesis is that, provided large training sets are available, morphometric parameters would suffice for unsupervised classification, overriding the need for examining other morphological features.

**Figure 1:**
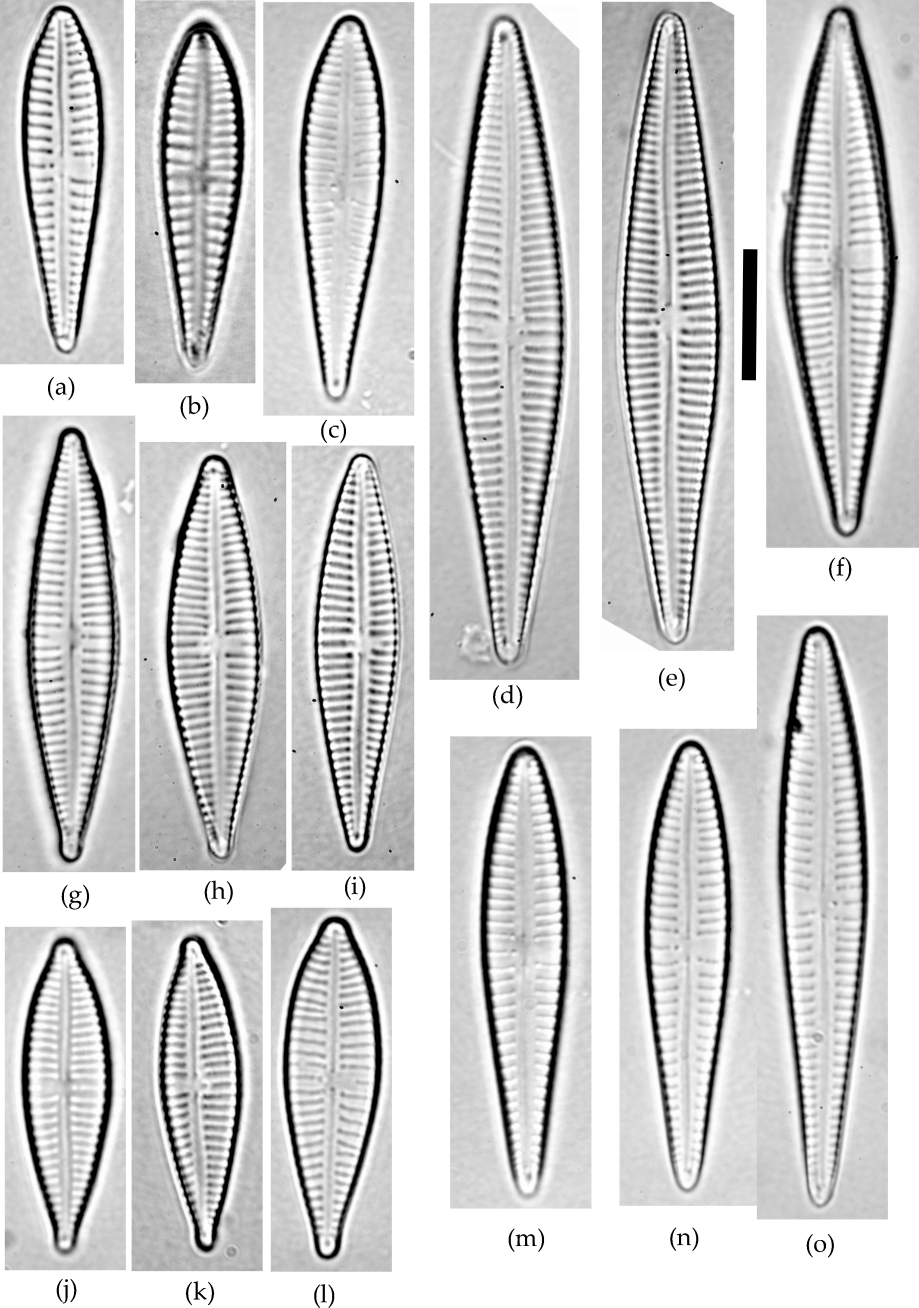
*Gomphonema* species analyzed in the study. (A–C) *G. jadwigiae*. (D–F) *G. gracile*. (G–I) *G. acidoclinatum*. (J–L) *G. parvulum*. (M–O) *G. auritum*. Scale bar = 10 µm.

## Materials & Methods

### Field and laboratory routines

A sample of epiphytic diatoms was collected from the surface of reed stems in Lake Villadangos (southeast León, northwest Spain, UTM 30T 272100 4711400) during July 2000. This an anthropic wetland located near Villadangos (León), and has a mean depth of 0.4 m, is 9.4 ha in and has 1.2 km perimeter, characterized by the presence of eutrophic waters. A detailed description of the wetland is available in Conty (2007). The sample was processed and analysed under light microscopy (LM) following standard protocols (CEN, 2003; CEN, 2004). Large populations of *Gomphonema* taxa dominated the diatom community, with 25 different species that were identified using a microscope (Leica DMRB, DIC 1,000×) according to usual reference works (Hofmann, Werum & Lange-Bertalot, 2011). All *Gomphonema* individuals lying in valve view (*N* = 523) were enumerated and photographed (Canon EOS400). Only five species attained large (*N* ≥ 45) populations (namely *G*. *gracile*, *G*. *auritum* A.Braun, *G*. *jadwigiae* Lange-Bert. and E.Reichardt, *G. acidoclinatum* Lange-Bert. and E.Reichardt and *G*. *parvulum*, Fig. 1), which were considered in subsequent analyses (*N* = 410). Morphometric parameters (length, width, length-width ratio and stria density, hereafter L, W, L/W and S respectively) were measured in each individual using Fiji software (Schindelin et al., 2012). *N* = 45 is the smallest sample size that represents the normal range (90%) of any population following a continuous distribution, with a 0.95 confidence. Original data are publicly available at FigShare (DOI: 10.6084/m9.figshare.4728406).

### Statistical analyses

Ten different numerical tools were tested and compared in this study. These algorithms were selected from among other many analogous methods proposed in the body of literature because (a) they are commonly available in statistical software applications, and (b) they have already been used in similar analyses.

1. Mixture analysis (MA): a maximum-likelihood distribution-based approach to test whether a variable distribution fits better in a mixture of (*a priori* defined) *n* normal overlapping distributions. The best-fit model can be chosen based on AIC values. Computations use the EM algorithm (Dempster, Laird & Rubin, 1977).

2. Canonical variates analysis (CVA): a discriminant analysis that evaluates quantitatively the distinctiveness of pre-classified groups of objects, estimating the spatial directions that maximize the differences between these groups. The output is a multivariate ordination plot that provides a linear combination of the classification variables having the highest possible multiple correlation with the selected groups. Computational details are available in Hammer, Harper & Ryan (2001).

3. Chi-squared automatic interaction detector (CHAID): a sophisticated segmentation modelling method for analysing large quantities of categorical data (Kass, 1980). It consists of a multivariate criterion-based algorithm that divides the test population into a number of distinct groups based on the categories of the most significant attribute. It uses the Chi-square test to determine the best next split at each step.

4. Random forests (RF): a nonparametric ensemble classification method that predicts classes based on the partition of input variables from multiple decision trees. The most reliable predictor is based on the decrease of classification accuracy when values of an attribute in a tree node are permuted randomly (Breman, 2001). Random forests produce lower prediction errors than other classification tree algorithms (Felde et al., 2014).

5. Boosted classification trees (BCT): a machine learning method which produces a prediction model in the form of an ensemble of decision trees. The algorithm uses a random sample of observations, builds independent sets of boosted trees for each category of the dependent variable, computes the predicted values for the observations in that sample, and fits a regression tree to the residuals, applying a logistic transformation to the predicted values before computing these residuals (Friedman, 2001).

6. k-means clustering (KMC): a nonhierarchical clustering method that searches for the partition of a sample into an *a priori* given number of groups so that the within-group sum of squares is minimal (Hartigan, 1975).

7. Expectation-maximisation (EM): a similar method that performs clustering by fitting a mixture of *n* different distributions to the data. The algorithm estimates the means and standard deviations for each cluster so as to maximise the likelihood of the observed input data (Witten & Frank, 2005).

8. Support vector machine (SVM): a learning algorithm that uses a hypothesis space of linear functions in a high-dimensional feature space, trained with a learning algorithm from optimization theory. It attempts to minimize the upper bound on the generalization error based on the principle of structural risk minimization (Hongzong et al., 2007).

9. Naïve Bayes Classifier (NBC): a simple probabilistic classifier where a single node, which represents a classification variable, is connected to all other nodes that represent predictor variables. The method is based on Bayesian theory with strong independence assumptions that the presence/absence of a particular feature of a class is not related to the presence/absence of any other feature (Liu et al., 2011).

10. K-nearest neighbour (KNN): a simple, nonparametric classifier in which the class of each object is determined with respect to the classes assigned to the nearest *k* objects, that is, it is specified according to the most repeated labels of these *k* objects (Han et al., 2015).

Computations and graphical outputs were performed with PAST v. 3.14 (Hammer, Harper & Ryan, 2001), Statistica v 10 (StatSoft, Tulsa, OK, USA: http://www.statsoft.com) and R (R Core Team, 2016) under RStudio (RStudio Team, 2015) with the Caret (Wing et al., 2016) and Ellipse (Murdoch & Chow, 2013) packages.

## Results

**Figure 2:**
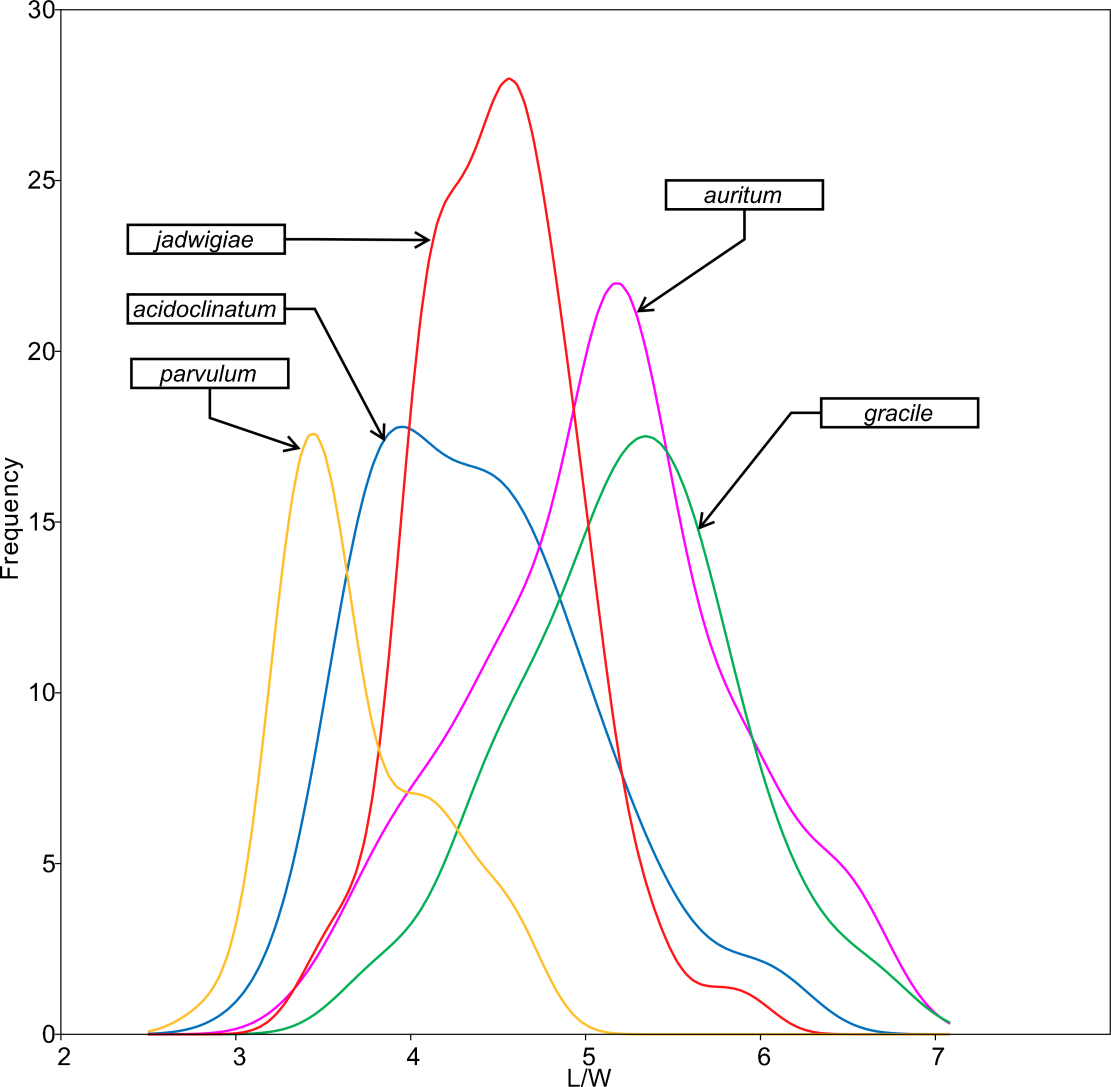
L/W histogram for the analysed Gomphonema populations: *G. jadwigiae* (*n* = 94), *G. acidoclinatum* (*n* = 89), *G. auritum* (*n* = 101), *G. gracile* (*n* = 80) and *G. parvulum* (*n* = 46). Data fitted to kernel distribution estimators.

**Figure 3:**
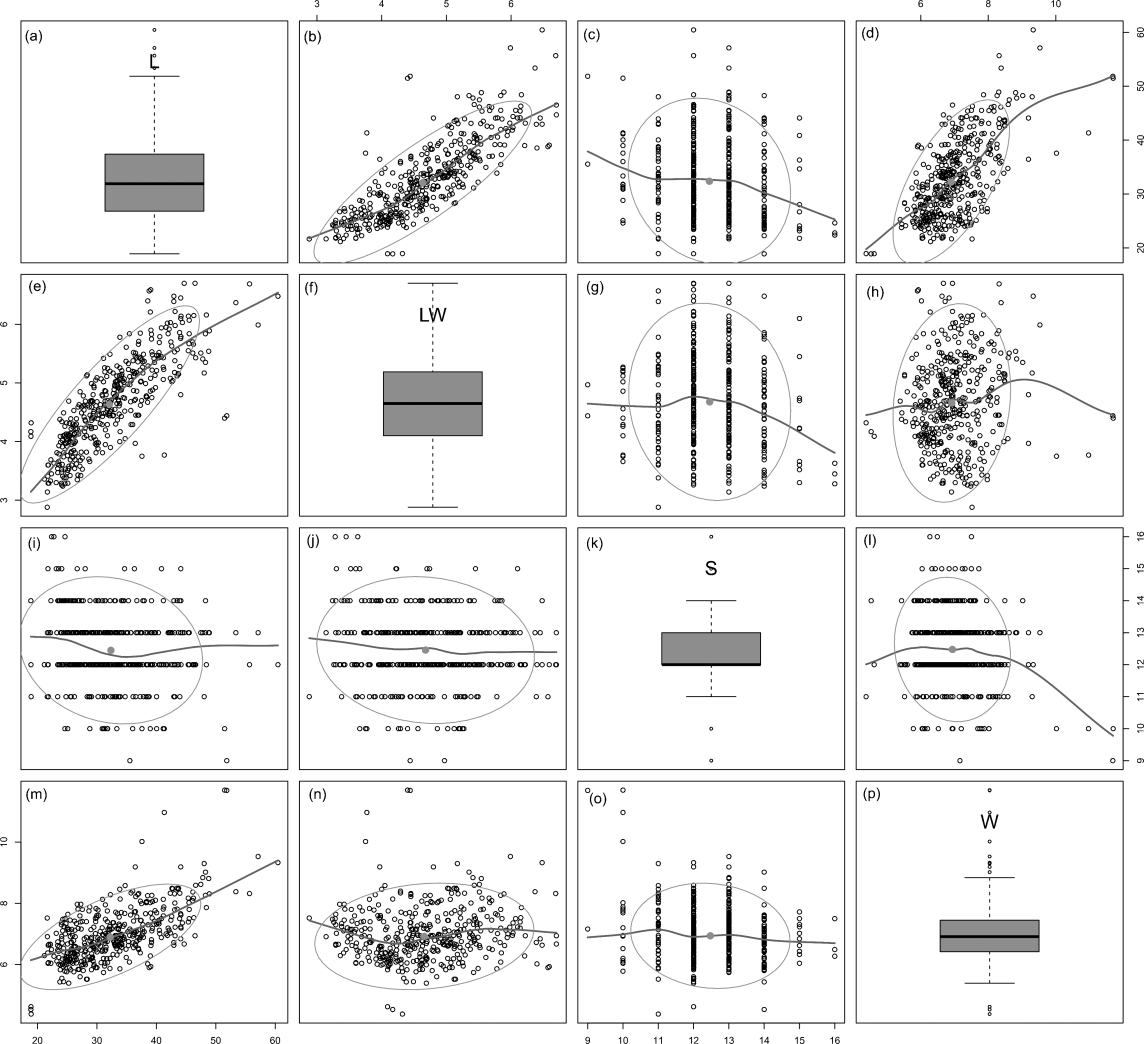
Scatterplot matrix of correlations between analysed morphometric parameters (A–P). Data fitted to LOESS smoothers and 95% confidence ellipses.

All analysed morphometric parameters overlapped between the species considered (e.g., L/W, Fig. 2). The lowest variability in terms of L, L/W and S parameters was found in *G*. *jadwigiae* (CV of 11.9%, 6.2% and 9.9%, respectively), while the least variable population in cell width was that of *G*. *auritum* (7.5%). Most parameters evaluated showed positive skewness, indicating right-tailed distributions. None of the variables examined in any of the *Gomphonema* species followed Gaussian distributions (Shapiro–Wilk test, *p* < 0.05). As expected, the L/W relationship was monotonic positive for all populations (Fig. 3). Stria density did not correlate with any other morphometric parameter.

### MA

**Figure 4:**
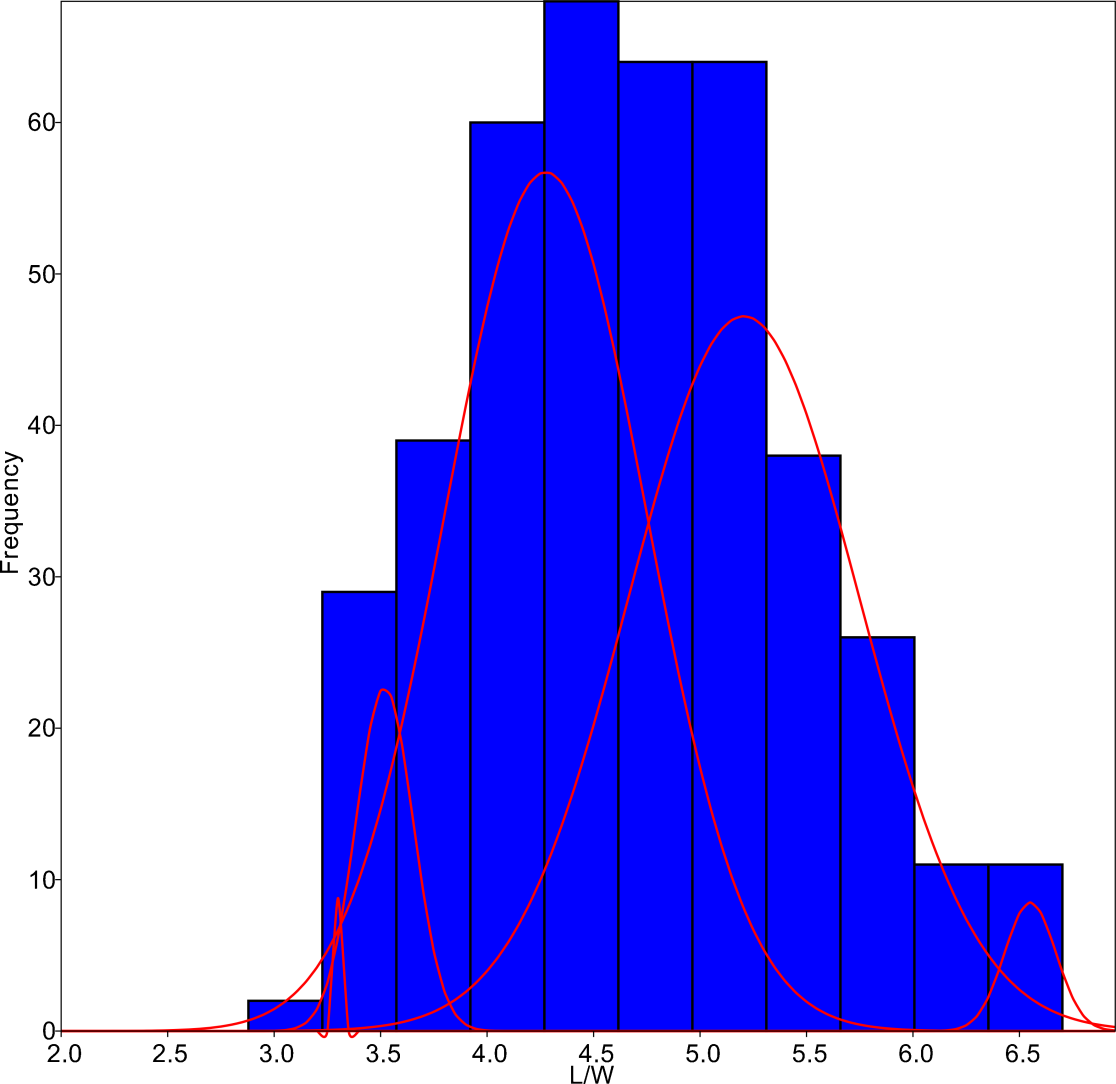
L/W histogram for the whole dataset and MA distributions adjusted to five classes (red curves).

The algorithm was set to fit five different populations (Fig. 4), although the lowest AIC values were found forcing 6–7 groups. Since MA is a univariate method, it provided four different classifications according to each parameter tested. On average, only 59.8% of the individuals were correctly classified by the MA method, with the highest success for *G*. *jadwigiae* (67.0%) and the lowest success for *G*. *parvulum* (40.0%). The best average results were obtained with W parameter (74.3%), while S had the lowest predictive power (43.8%).

### CVA

**Figure 5:**
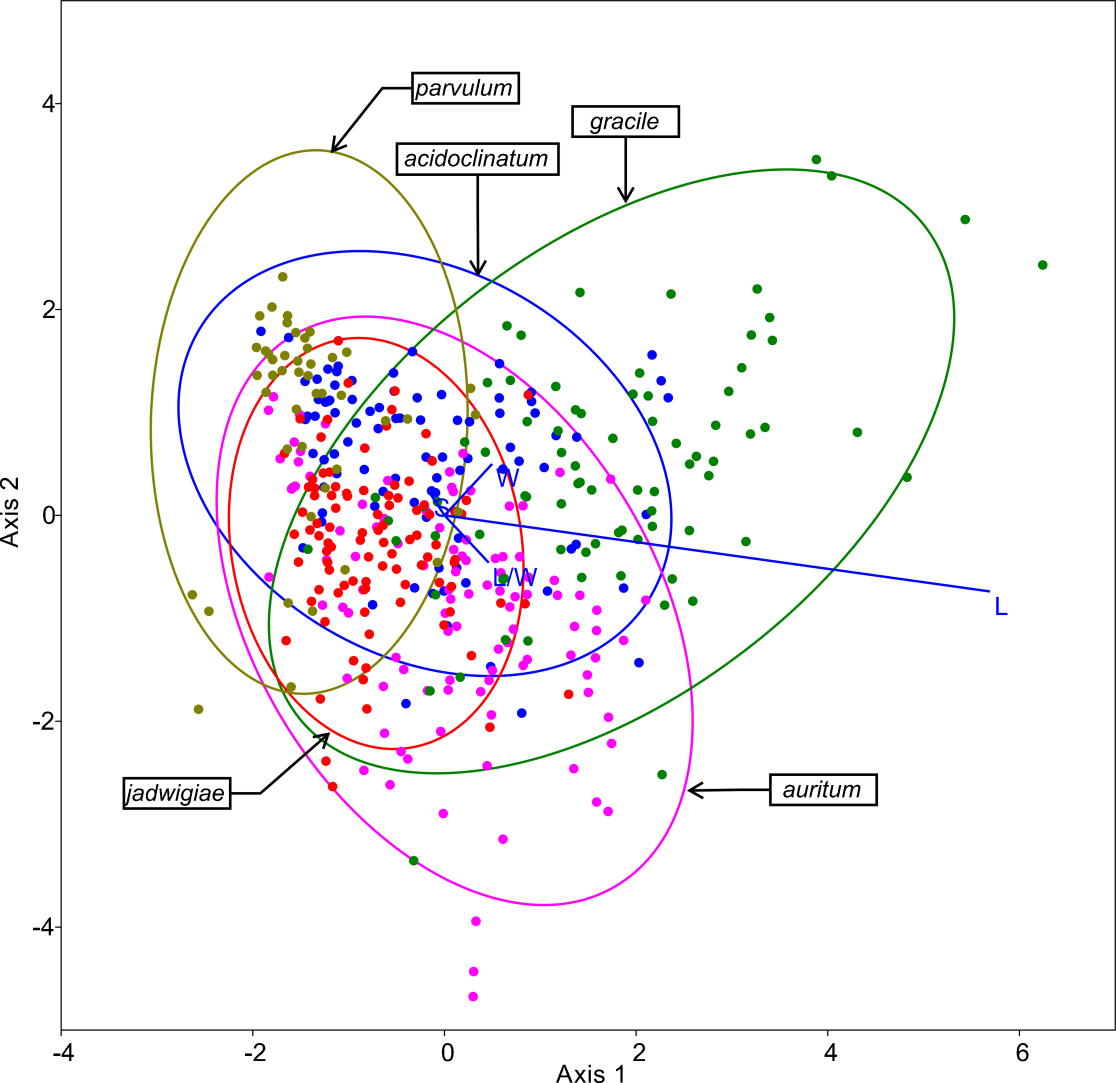
CVA ordination biplot, dots represent individuals and line predictor variables. Points fitted to 95% confidence ellipses.

The resulting ordination of this method is shown in Fig. 5, highlighting cell length as the most important variable for specimen classification. The species most frequently misidentified according to this algorithm was *G*. *acidoclinatum* (only 33.7% of correctassignations), whereby 29.2% of cases were identified erroneously as *G*. *parvulum*. On the contrary, *G*. *gracile* was correctly classified in 68.8% of cases. In total, 55.1% of items were correctly classified.

### CHAID

**Figure 6:**
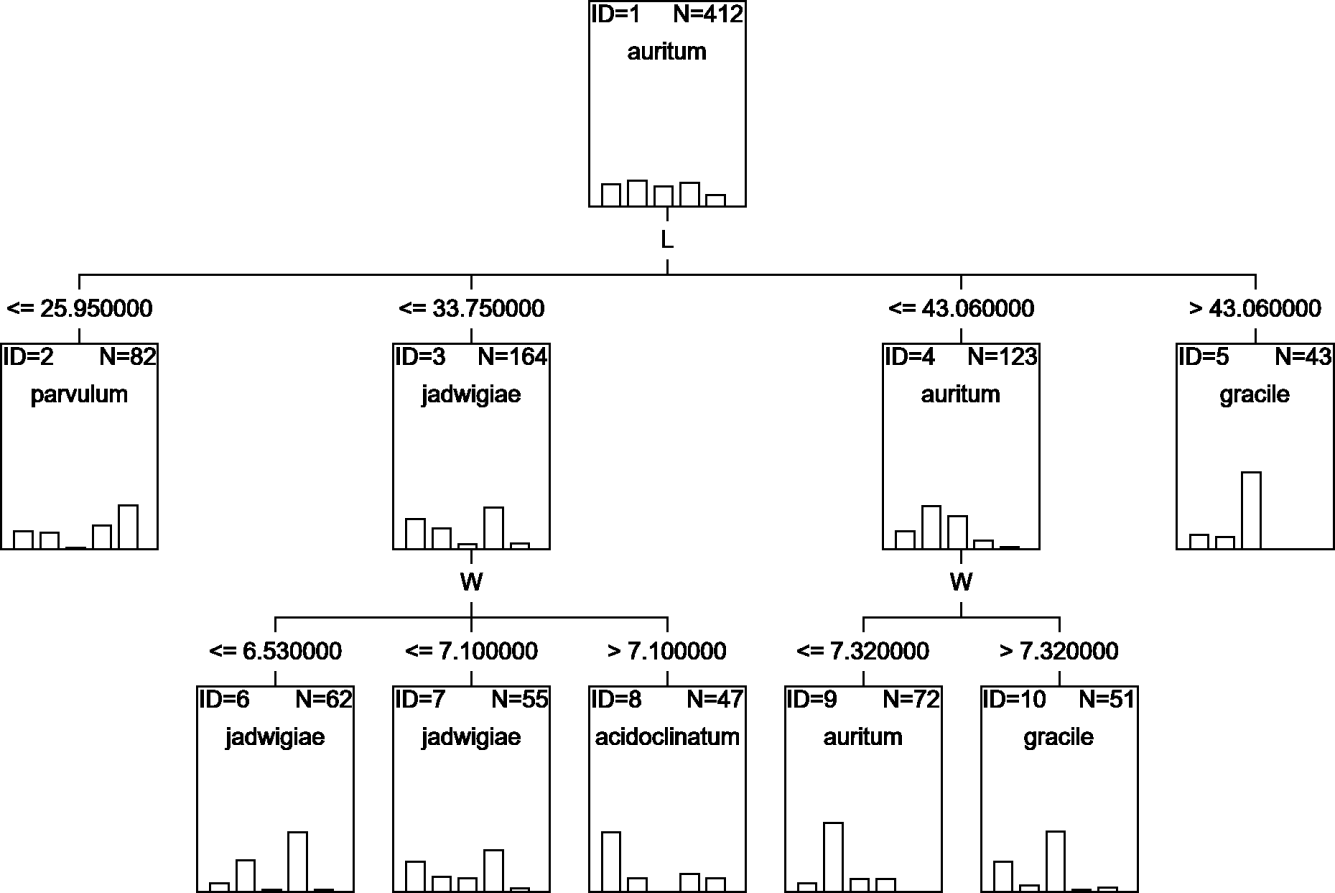
CHAID classification tree.

A classification tree using only L and W parameters as classifying variables can be drawn (Fig. 6), but with an estimated classification risk of 44.2 ± 0.02%. According to the resulting confusion matrix (data not shown), as in the case of CVA results, *G. acidoclinatum* was the taxon most often misidentified (in 30.4% of cases), frequently (in 23.6% of cases) as *G*. *jadwigiae*, whereas *G*. *gracile* was again correctly classified in 77.5% of cases. Resulting classification used only L and W variables.

### RF

**Figure 7:**
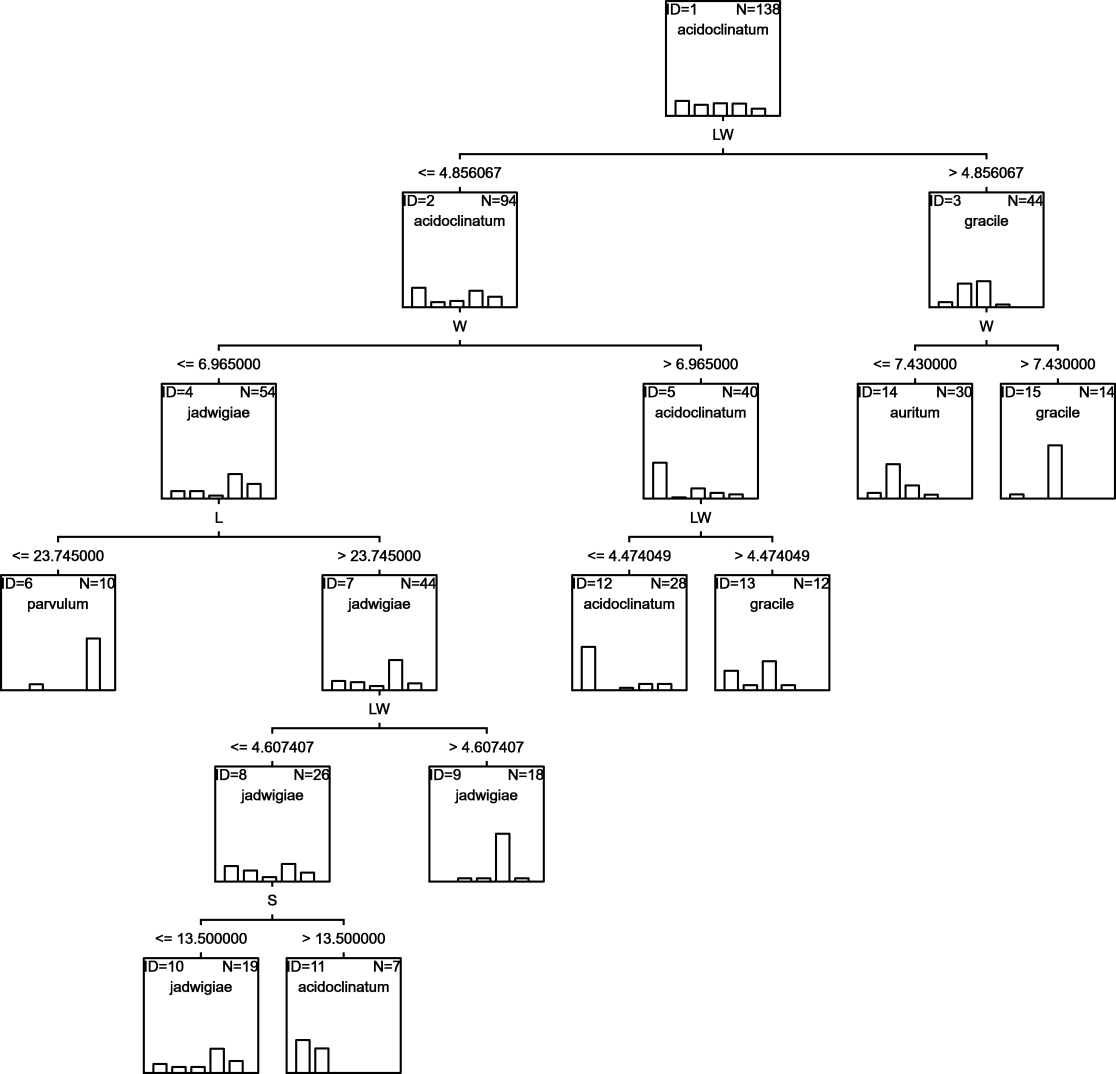
RF classification tree.

Random forests algorithm selected the classification tree shown in Fig. 7 as the most parsimonious among the other 200 models tested. This led to a success percentage in correct assignations of 61.7%. The most important classificatory variable was L. Most misidentifications affected *G*. *acidoclinatum* (50.0%), erroneously considered *G*. *jadwigiae* in 21.9% of cases. In this algorithm, the taxon achieving maximal correct assignations (68.3%) was *G*. *jadwigiae*.

### BCT

The classificatory success of this method is 67.9%. As in the case of RF, the most important classificatory variable was L. Most frequent misclassifications were observed again in *G*. *acidoclinatum* (45.2%), frequently (16.1%) assigned to *G*. *jadwigiae*. *Gomphonema gracile* was correctly identified in 77.1% of cases.

### KMC

K-means clustering forced on 5 groups achieved an average of 70.2% of correct classifications. While taxa such as *G*. *acidoclinatum* or *G*. *jadwigiae* were always (100%) assigned to unique clusters, the algorithm failed to discriminate *G*. *auritum* and *G*. *gracile*, which were gathered in the same cluster in 81% of cases. *Gomphonema parvulum* was also misidentified in 48.9% of cases.

### EM

The success percentage of this method was only 44.1%. As in the case of KMC, two different class objects (*G*. *acidoclinatum* and *G*. *parvulum*) were erroneously gathered together. The identification success ranked from 44.3% (*G*. *acidoclinatum*) to 71.3% (*G*. *jadwigiae*).

### SVM

A total of 60.2% specimens were correctly classified by this algorithm. The species most often misidentified was *G*. *parvulum* (50.0%, of which 40.0% were misidentified as *G. jadwigiae*), while 71.4% of *G. gracile* individuals tested were correctly identified.

### NBC

A total of 62.1% specimens were correctly classified by this algorithm. The species most often misidentified was *G*. *acidoclinatum* (71.4%, of which in 23.8% of cases misidentified as *G*. *parvulum*), while 76.9% of the *G*. *jadwigae* individuals tested were correctly identified.

### KNN

The predictive power of this method was only 49.5%. The percentage of correct classifications ranked from 61.9% (*G*. *gracile*) to 30.0% (*G*. *parvulum*, in 40.0% of cases misidentified as *G*. *auritum*).

## Discussion

During recent decades, quantitative techniques have proposed many different methods to suggest classifications of organisms based on metric characters (Julius et al., 1998). Currently, size analysis is a potentially powerful tool for understanding diatom community dynamics and systematic relationships (Spaulding et al., 2012). Our study tested different classification algorithms based on metric and meristic parameters that are commonly recorded in diatom taxonomy, and which have proven to be useful to segregate morphologically similar taxa. For instance, Paull et al. (2008) demonstrated using linear discriminant analysis that two closely related *Staurosirella* species could be distinguishedwith an error of 6% using only cell width and areolae length. In the genus *Kobayasiella*, a single morphometric character can separate all individuals from sister species (Buczkó, Wojtal & Jahn, 2009).

Our results show that a majority of methods selected L as the best classificatory parameter, in contrast with previous studies showing S and W as the most stable characters (Genkal, 2004). Particularly, S is known to change little with changing valve length, although it may vary due to environmental factors (Cox, 2010). On the contrary, S has been found to be inadequate to distinguish diatom species in other works (Paull et al., 2008). With respect to L/W parameter, this is the most popular ratio in algal taxonomy, and is commonly regarded as a reliable parameter, despite the fact that it is often size-dependent, compounding variations from several sources (Theriot, 1988).

Multivariate methods such as cluster or classificatory algorithms have started to be adopted in diatom taxonomy (Buczkó, Wojtal & Jahn, 2009). These techniques are, in contrast to the classical methods, more robust when dealing with complex multivariate data (Felde et al., 2014). Both cluster analysis and ordination techniques have similar aims in that they attempt to explore multivariate datasets by reducing their dimensionality and summarising the major patterns of variation within the datasets (Felde et al., 2014). In our analyses, cluster methods (KMC, MA, KNN, EM) performed somewhat worse than classification algorithms (average classification risk: 44.1% *vs*. 39.5%, Table 1). Although the computational requirements are much lower in clustering techniques, the assignation of objects to each cluster must be supervised *a posteriori* (in this case, by calculating the modal class of each cluster). The best results (70.2% correct identification) were obtained by the relatively simple KMC, but the outcome of this algorithm suggested the presence of only 4 clusters. The Calinsky and silhouette criteria confirm that optimal partitioning of data is obtained for *n* = 4. The confusion matrix shows that the algorithm gathered *G .acidoclinatum* and *G*. *parvulum* in the same group, and this misidentification accounted for the largest amountof the classification failure observed. The MA method also indicated a different number of taxa in the studied assemblage according to AIC values, although the statistically optimum number determined by AIC may not be particularly useful (Felde et al., 2014). Within classification methods, best results were obtained by BCT (67.9%), and this method has proven to provide more accurate results when compared to other tree-based classification techniques (Bauer & Kohavi, 1999; Austin & Lee, 2011).

**Table 1:** Percentage of correct classifications achieved by each method.

Method	Correct classifications (%)
^*^KMC	70.2
BCT	67.9
NBC	62.1
RF	61.7
SVM	60.2
^*^MA	59.8
CHAID	55.8
CVA	55.1
^*^KNN	49.5
^*^EM	44.1

**Notes.**

* Cluster methods.

Contrary to our expectations, classification success was unrelated to sample size (number of items per class). The taxa that were most oten correctly classified and less often misclassified (*G*. *acidoclinatum* and *G*. *gracile*, respectively) were not the species with the largest or lowest numbers of individuals. Classification risk was also independent of the variability of each species (measured as CV in L, W, L/W and S). This suggests that unsupervised classification based on metric parameters may lead to consistent results independently of the descriptive statistics of the groups involved.

Notwithstanding, the best scores achieved by the methods analysed in this study are far below other automated identification techniques that consider not only morphometry, but also cell shape and structural features, even when tested over similar species. For instance, SHERPA obtained classification accuracies ranging from 98.9% to 100.0% when applied to the *Sellaphora pupula* complex (Kloster, Kauer & Beszteri, 2014). Similarly, the system developed in the ADIAC project allowed the identification of 37 species with an accuracy of 97% (Buf & Bayer, 2002). Similar success ratios have been reported using a multi-label classification system for diatom image classification developed by Dimitrovski et al. (2012). This shows that the biological relevance of the morphological distinctness of diatoms depends on whether the differences can be explained simply by size differences (Beszteri, Ács & Medlin, 2005), and otherwise shape analyses allows the segregation of groups that have different size ranges but vary only subtly in other characters (Mou & Stoermer, 1992). When shape group separation is not evident, morphometric measures such as L, W or S may be used (Kingston & Pappas, 2009).

## Conclusions

In the light of our results, we cannot recommend the exclusive use of morphometric measurements for unsupervised diatom classification that aims at segregating morphologically similar species. According to our results and the available literature, the combined use of morphometry and morphology seems to best suit this purpose.

## Abbreviations

BCT: Boosted classification trees
CHAID: Chi-squared automatic interaction detector
CVA: Canonical variates analysis
EM: Expectation-maximisation
KMC: K-means clustering
KNN: K-nearest neighbour
MA: Mixture analysis
NBC: Naïve Bayes Classifier
RF: Random forests
SVM: Support vector machine

## Additional Information and Declarations

### Data Availability

The following information was supplied regarding data availability: Blanco, Saul (2017): Blanco et al Gomphonema.csv. figshare.
https://dx.doi.org/10.6084/m9.figshare.4728406.v1.
